# Activated Leukocyte Cell Adhesion Molecule (ALCAM), a Potential ‘Seed’ and ‘Soil’ Receptor in the Peritoneal Metastasis of Gastrointestinal Cancers

**DOI:** 10.3390/ijms24010876

**Published:** 2023-01-03

**Authors:** Yi Ming Yang, Lin Ye, Fiona Ruge, Ziqian Fang, Ke Ji, Andrew J. Sanders, Shuqin Jia, Chunyi Hao, Q. Ping Dou, Jiafu Ji, Wen G. Jiang

**Affiliations:** 1 Cardiff China Medical Research Collaborative, Division of Cancer and Genetics, University School of Medicine, Heath Park, Cardiff CF14 4XN, UK; 2 Gastrointestinal Cancer Center, Key Laboratory of Carcinogenesis and Translational Research (Ministry of Education), Peking University Cancer Hospital and Institute, Fucheng Street, Haidian District, Beijing 100089, China; 3 School of Natural and Social Science, University of Gloucestershire, Francis Close Hall, Swindon Road, Cheltenham GL50 4AZ, UK; 4 Barbara Ann Karmanos Cancer Institute, Departments of Oncology, Pharmacology and Pathology, School of Medicine, Wayne State University, Detroit, MI 48201, USA

**Keywords:** ALCAM, CD166, mesothelial cells, gastric cancer, pancreatic cancer, peritoneal metastasis, transcoelomic metastasis, tumour-mesothelial interaction, SRC kinase

## Abstract

Activated Leukocyte Cell Adhesion Molecule (ALCAM/CD166) is a cell–cell adhesion protein conferring heterotypic and homotypic interactions between cells of the same type and different types. It is aberrantly expressed in various cancer types and has been shown to be a regulator of cancer metastasis. In the present study, we investigated potential roles of ALCAM in the peritoneal transcoelomic metastasis in gastrointestinal cancers, a metastatic type commonly occurred in gastro-intestinal and gynaecological malignancies and resulting in poor clinical outcomes. Specifically, we studied whether ALCAM acts as both a ‘seed’ receptor in these tumour cells and a ‘soil’ receptor in peritoneal mesothelial cells during cancer metastasis. Gastric cancer and pancreatic cancer tissues with or without peritoneal metastasis were compared for their levels of ALCAM expression. The impact of ALCAM expression in these tumours was also correlated to the patients’ clinical outcomes, namely peritoneal metastasis-free survival. In addition, cancer cells of gastric and pancreatic origins were used to create cell models with decreased or increased levels of ALCAM expression by genetic knocking down or overexpression, respectively. Human peritoneal mesothelial cells were also genetically transfected to generate cell models with different profiles of ALCAM expression. These cell models were used in the tumour-mesothelial interaction assay to assess if and how the interaction was influenced by ALCAM. Both gastric and pancreatic tumour tissues from patients who developed peritoneal metastases had higher levels of ALCAM transcript than those without. Patients who had tumours with high levels of ALCAM had a much shorter peritoneal metastasis free survival compared with those who had low ALCAM expression (*p* = 0.006). ALCAM knockdown of the mesothelial cell line MET5A rendered the cells with reduced interaction with both gastric cancer cells and pancreatic cancer cells. Likewise, levels of ALCAM in both human gastric and pancreatic cancer cells were also a determining factor for their adhesiveness to mesothelial cells, a process that was likely to be triggered the phosphorylation of the SRC kinase. A soluble ALCAM (sALCAM) was found to be able to inhibit the adhesiveness between cancer cells and mesothelial cells, mechanistically behaving like a SRC kinase inhibitor. ALCAM is an indicator of peritoneal metastasis in both gastric and pancreatic cancer patients. It acts as not only a potential peritoneal ‘soil’ receptor of tumour seeding but also a ‘soil’ receptor in peritoneal mesothelial cells during cancer metastasis. These findings have an important therapeutic implication for treating peritoneal transcoelomic metastases.

## 1. Introduction

Peritoneal metastases, also referred to as peritoneal transcoelomic metastasis, peritoneal carcinomatosis, or secondary peritoneal surface malignancies, are commonly seen in patients with primary cancers of the intraperitoneal origin including gynecological, gastrointestinal and pancreatic cancers [1,2,3,4,5,6,7]. Ovarian cancer has the highest incidence of peritoneal metastasis which can be as high as 70% of the patients [8]; other important contributors to peritoneal metastasis include gastric cancer (14% developing peritoneal metastasis) [9], pancreatic cancer (13.5%) [10] and colorectal cancer (4–15%) [11,12], whereas peritoneal metastasis for tumours outside the peritoneal cavity are less common and mostly of these are from breast cancer and lung cancer [7,13]. Tumours with peritoneal seeding are usually diagnosed at stage IV and sadly, patients with wide spread transcoelomic spreading survive no longer than 6 months [14]. Therapeutic options with efficacy are limited for such patients [15,16,17,18,19,20]. With the aid of washing cytology and staging laparoscopy, more and more peritoneal metastases are diagnosed at the occult stage. Clinically, locally advanced cancers are those patients with only intraperitoneal free cancer cells but without visible peritoneal seeding and their outcome is more favorable [21,22,23] than the later stages, namely stage III [24,25,26,27] and stage IV cancers [28,29]. In the National Comprehensive Cancer Network (NCCN) guidelines, positive peritoneal washing cytology is categorized as distant metastasis for both gastric and ovarian cancers. Treatment option for peritoneal metastasis is rather limited. Hyperthermic intraperitoneal chemotherapy (HIPEC) and intraperitoneal chemotherapy (IPEC) are adopted as prophylactic methods that could improve the prognosis of this group of patients [5,6,25,30]. In combination with peritoneal irrigation, the occult stage IV patients with gastric cancer likely to have a longer survival time [31]. Therefore, additional therapeutic methods are needed to further enhance the efficacy of currently available therapeutic strategies for patients with occult peritoneal metastasis.

The ‘seed and soil’ theory was proposed as a mechanism of metastatic diseases some 130 years ago by Stephen Paget [32]. Although the initial description by Paget was mainly on bone and liver metastases in which the microenvironment in these organs/tissues (soil) make them more receptable to particular type of cancer cells (seeds), the past century has seen a great deal more understanding into the ‘seed and soil’ theory. Some of the organs and tissues are commonly known as the rich ‘soil’ to receive cancer cells, such as bone, liver, brain and lung. Over the decades, a number of molecules have been identified as ‘soil receptors’ for the target organs/tissues and ‘seed receptors’ for spreading cancer cells. The commonly recognized ones include CD44 as a seed receptor and galectins and hyaluronic acid (HA) as soil receptors [33,34,35]; SSSRs (S100 soil sensor receptors including S100/A8 and A9) [36], and a few other cell adhesion molecules, including ICAMs as ‘soil’ or ‘seed’ receptors. These ‘seed and soil’ receptors have been shown to facilitate the spreading of cancer cells in order to identify their suitable destination to develop distant metastasis. However, given the complexity of cancer types and target organs, there appears to be a large gap here.

ALCAM is a transmembrane protein of the immunoglobulin superfamily of cell adhesion proteins, known for its role in mediating heterotypic and homotypic interactions [37]. ALCAM is linked to the cytoskeleton system via interacting with the ERM (ezrin-moesin-radixin) subcoat protein family, a process regulated in part by the SRC kinase. The heterotypic binding partners of ALCAM include CD6, L1CAM (also known as CD171 or NCAM-L1) and possibly CD9, CD34 and CD44 together with certain integrins [38,39]. However, the most interesting feature of this protein is the ALCAM-ALCAM-interaction-mediated homotypic interactions between different cells, and interaction of ALCAM to CD6 or L1CAM on immune cells [40]. It has been well demonstrated that ALCAM on cancer cells and ALCAM on endothelial cells can trigger ALCAM-ALCAM mediated cancer-endothelial interactions and lead to cancer cells’ clustering over the endothelium layer, thus orchestrating the extravasation process [41,42,43]. However, little is known whether ALCAM has a role in the peritoneal metastasis by mediating the interaction between cancer cells and peritoneal mesothelial cells. Mesothelial cells are epithelial cells by origin but have a unique feature of lining the peritoneal cavity as a protective layer to the cavity and preventing organs in the peritoneal cavity from adhering to each other. As stated earlier, peritoneal metastases are rather commonly for the primary cancers of the gastrointestinal and gynaecological origins. Thus, in this rich ‘soil’ for cancer metastases, it is quite possible that the ALCAM-ALCAM interaction between cancer and mesothelium may make ALCAM act as both a ‘seed’ receptor of cancer cells and a ‘soil’ receptor for the mesothelium.

Although there is very little knowledge on ALCAM in mesothelial cells, the role of ALCAM in mesothelial cell-derived malignancies, namely pleural mesothelioma, has been reported. A study by Inaguma et al. [44] showed that about one quarters of the pleural mesotheliomas expressed ALCAM and this expression is an independent prognostic indicator for the survival of the patients. In addition, soluble ALCAM was found to be able to inhibit ALCAM-mediated cell adhesion and migration of mesothelioma cells [45]. Studies on gastrointestinal cancers in the contact of peritoneal mesothelium are rather uncommon. It has been recently reported [46] that extracellular vesicles produced by colorectal and ovarian cancer cells contain ALCAM and this observation has a potential impact on extracellular vesicle-mediated immunosuppression in the patients. However, little is known about the role of ALCAM in peritoneal metastasis.

In the present study, we first explored if expression levels of ALCAM may have an impact on the peritoneal metastases of the patients with gastric and pancreatic cancers. Following showing a positive correlation in the clinical settings, we went to create cell models of gastric and pancreatic cancer cells as well as mesothelial cells with altered levels of ALCAM expression. Using these cell models, we studied the roles of ALCAM in cancer cells and mesothelial cells during metastasis and the interplay between these cells and disclosed the potential role the SRC kinase in this interaction.

## 2. Results

### 2.1. ALCAM in Tumours Which Developed Peritoneal Metastasis 

**Figure 1:**
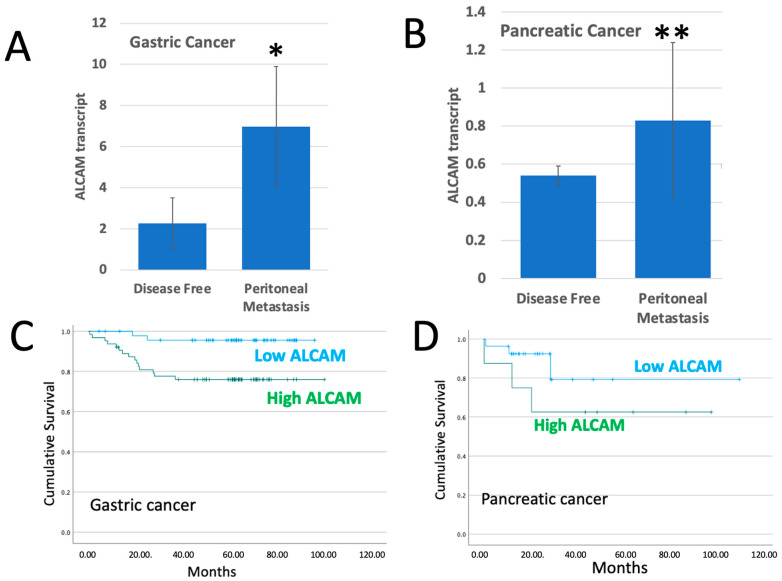
ALCAM transcript expression in tumours (**A**,**B**) and in relationship with peritoneal metastasis free survivals (**C**,**D**). (**A**,**B**): The ALCAM levels were compared between tumours from patients who remained disease-free and those who developed peritoneal metastases (*n* = 18 with peritoneal metastasis and *n* = 112 who were disease free for the gastric cancer group and *n* = 6 and *n* = 35 for the pancreatic cancer group). * *p* = 0.037, ** *p* = 0.01. (**C**,**D**): Gastric cancer patients with high levels of ALCAM had a significantly shorter survival than those with low levels (*p* = 0.006). A similar shorter survival with seen with pancreatic cancer patients although this is yet to reach statistical significance (*p* = 0.208).

Our gastric clinical cohort recorded 18 patients who developed peritoneal metastasis and our pancreatic cohort recorded 6 patients with peritoneal metastasis. We compared the ALCAM transcript expression levels between the patients with peritoneal metastasis and the patients who remained uneventful (*n* = 112 for the gastric cohort and *n* = 35 for the pancreatic cohort). As shown in Figure 1, patients who had peritoneal metastases had significantly higher levels of ALCAM than those without (*p* = 0.037 for gastric cancer (Figure 1A) and *p* = 0.01 for pancreatic cancer (Figure 1B)). 

Next, we analyzed if the levels of ALCAM had a relationship with the peritoneal metastasis related survival of patients with gastric and pancreatic cancer. As shown in Figure 1C,D and Table 1, significantly shorter peritoneal metastasis-free survival was seen in gastric cancer patients with high levels of ALCAM (*p* = 0.006). A similar shorter survival was also seen in patients with pancreatic cancer, although the difference was not statistically significant (*p* = 0.208), owing to the smaller number of the patients in this cohort.

### 2.2. The Creation of Cell Models with Altered Levels of ALCAM 

**Figure 2:**
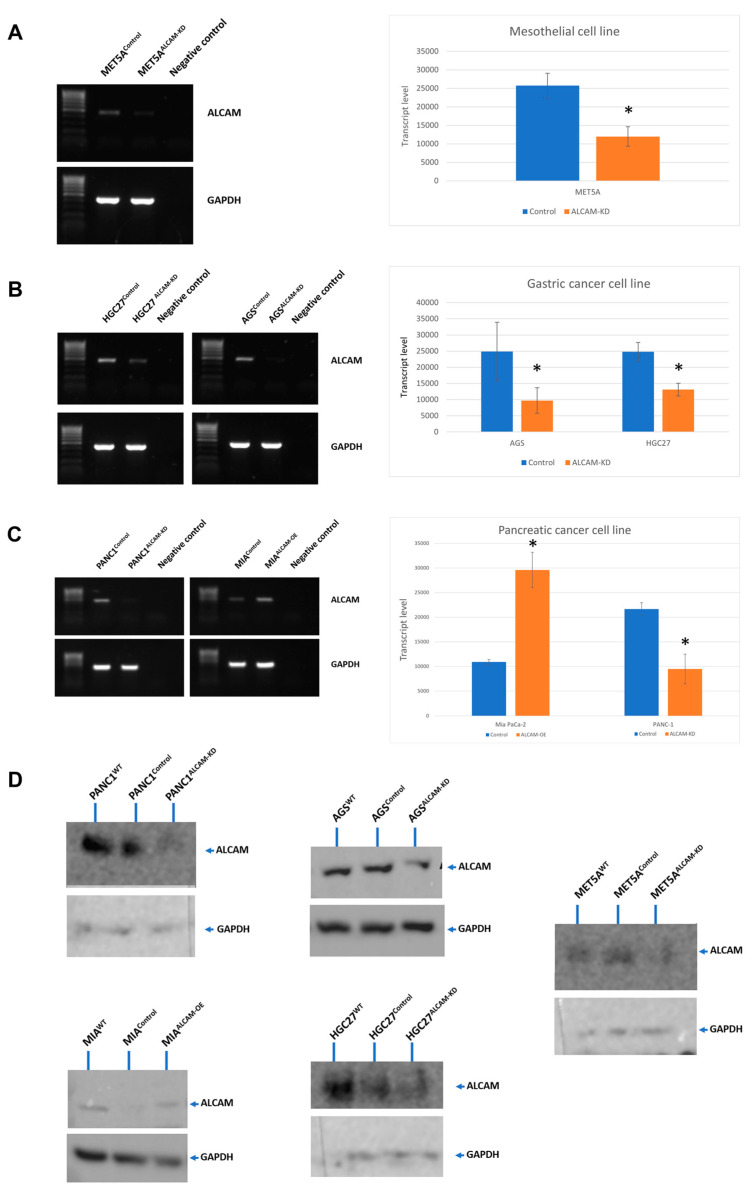
Creation of cell models with differential expression of ALCAM as confirmed by PCR (**left panel**) and quantitative PCR (**right panel**). (**A**): The ALCAM transcript expression in MET5A control (MET5A^Control^) and ALCAM knockdown cells (MET5A^ALCAM-KD^). (**B**): The ALCAM transcript expression levels in HGC-27 control (HGC27^Control^) and ALCAM knockdown cells (HGC27^ALCAM-KD^) (**left**), as well as AGS control (AGS ^Control^) and ALCAM knockdown cells (AGS^ALCAM-KD^) (**right**). (**C**): The ALCAM transcript expression levels in PANC-1 Control (PANC1^Control^) and ALCAM knockdown cells (PANC1^ALCAM-KD^) (**left**), as well as MIA PaCa-2 Control (MIA^Control^) and ALCAM overexpression cells (MIA^ALCAM-OE^) (**right**). *: ALCAM-modified cell lines which showed significant changes compared with their respective control cells (*p* < 0.05). (**D**): Knockdown of ALCAM protein in the respective cell lines as shown by protein blotting.

The above clinical findings reveal that gastric and pancreatic cancer patients with peritoneal metastasis had significantly higher ALCAM expression and the peritoneal metastasis-free survival of the patients was shorter in high ALCAM expression group compared with low ALCAM expression group. To validate the potential promoting role of ALCAM in terms of peritoneal metastasis, we created ALCAM-manipulated cell models in both cancer cells and mesothelial cells to explore the tumour-mesothelial cell interaction in vitro. Gastric cancer cell lines HGC-27 and AGS, pancreatic cancer cell line PANC-1 and mesothelial cell line MET5A were used to generate ALCAM knockdown cell models because of their relatively higher ALCAM expression. The pancreatic cancer cell line MIA PaCa-2 showed relatively lower ALCAM expression, thus it was used to create ALCAM overexpression cell model. As demonstrated in Figure 2, the ALCAM expression of each cell line showed a clear change following transfection, as detected by PCR, qPCR and by protein blotting.

### 2.3. Dynamic Monitoring of Gastric Tumour-Mesothelial Interaction Assocaited with ALCAM Level Alteration

**Figure 3:**
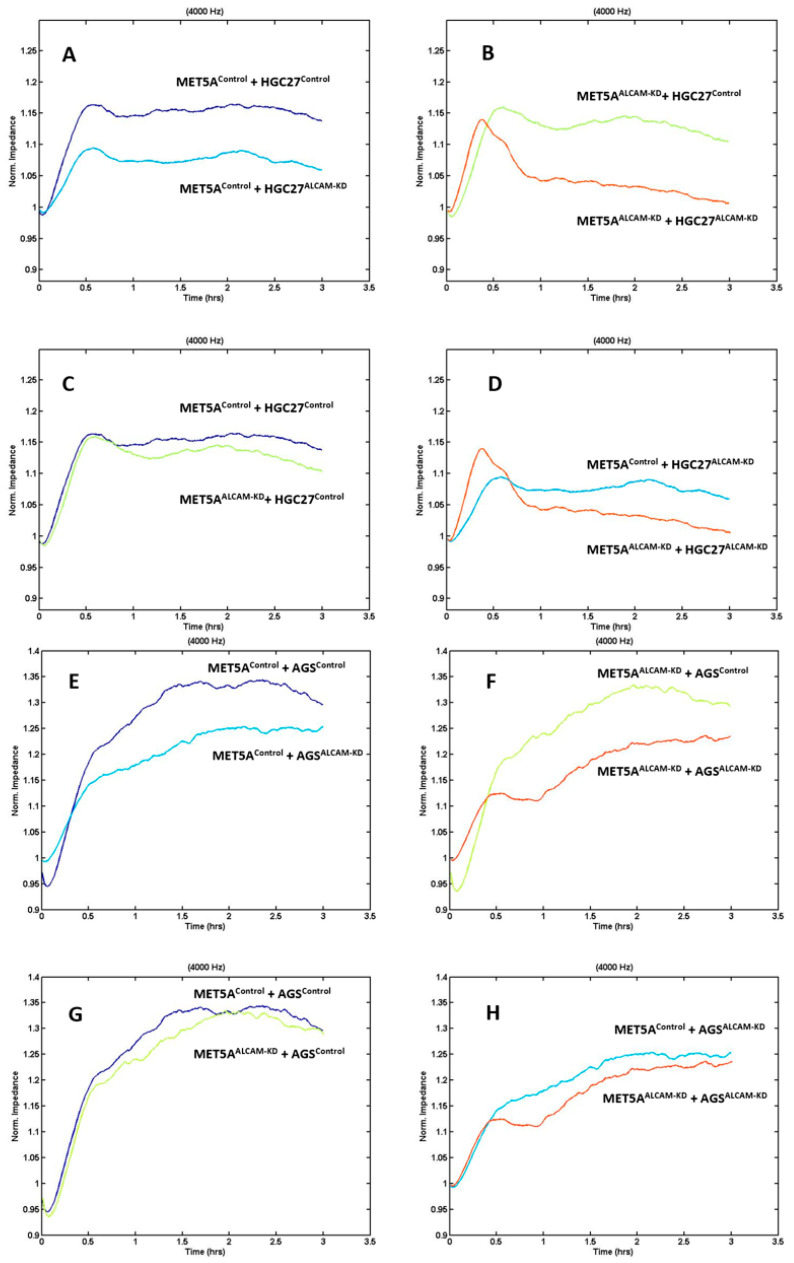
ECIS based evaluation of gastric adhesion to mesothelial cells. (**A**–**D**): Adhesion of HGC27 cells to MET5A mesothelial cells; (**E**–**H**): Adhesion of AGS cells to MET5A cells; Shown are monitoring at 4000 Hz. MET5A^Control^, AGS^Control^, HGC27^Control^: control transfected cells; MET5A^ALCAM-KD^, AGS^ALCAM-KD^, HGC27^ALCAM-KD^: cells with ALCAM knockdown by way of cell transfection. Replicate *n* = 4.

**Figure 4:**
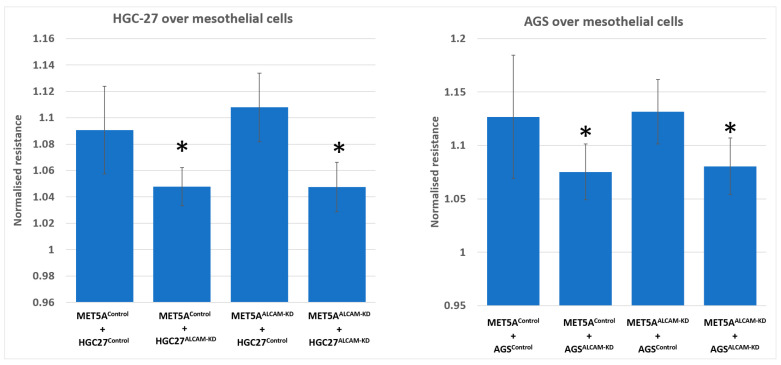
Interaction between HGC-27 (**left**) or AGS (**right**) gastric cancer cells and MET5A mesothelial cells. Both gastric cancer cell lines after knocking down ALCAM showed reduced adhesion to MET5A mesothelial cells. * Groups of cells with ALCAM knockdown compared with the groups of control MET5A cells plus control cancer cells (*p* < 0.05, replicate *n* = 4).

We created several cell models including MET5A mesothelial cells with ALCAM knockdown, AGS and HGC27 gastric cancer cells with ALCAM knockdown, PANC-1 pancreatic cancer cells with ALCAM knockdown and MIA PaCa-2 cells with ALCAM overexpression. To monitor if the changes of ALCAM in these cells impacted the interaction between cancer cells and mesothelial cells, we used the electric cell-substrate sensing (ECIS) as a method to trace the dynamic interactions between the two cell types. Gastric cancer control cells which expressed high levels of ALCAM adhered rapidly to control MET5A cells which also expressed high levels of ALCAM (Figure 3A for HGC27 and Figure 3E for AGS). However, following knocking down ALCAM in both gastric cancer cell lines, the pace of adhesiveness to control MET5A were substantially reduced (Figure 3A,E). Additionally, knocking down ALCAM from MET5A cells resulted in a marginal reduction of adhesion by control gastric cancer cells (Figure 3C for HGC27 and Figure 3G for AGS). More importantly, when ALCAM was knocked down from both mesothelial cells and gastric cancer cells, the interaction between the two cell types were greatly reduced compared to individual knockdown cells (Figure 3B,D,F,H). Figure 3 showed the electric resistance value of each ECIS experimental group 1 h after cancer cells were added. It is clear that adhesion of gastric ALCAM knockdown cells to mesothelial cells, control or ALCAM knockdown, was significantly reduced (Figure 4).

### 2.4. Dynamic Monitoring of Pancreatic Tumour-Mesothelial Interactions Associated with ALCAM Alterations

**Figure 5:**
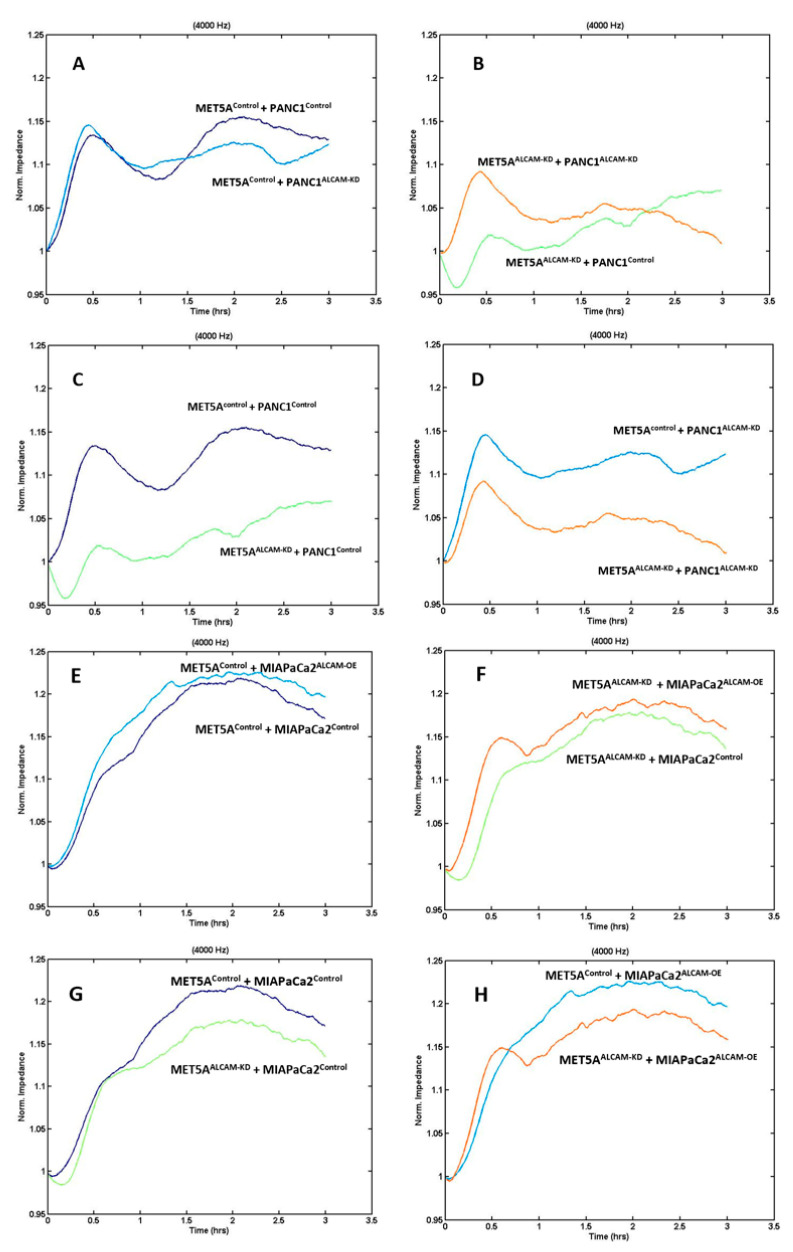
ECIS based evaluation of pancreatic adhesion to mesothelial cells. (**A**–**D**): Adhesion of PANC1 cells to MET5A mesothelial cells; (**E**–**H**): Adhesion of MIA PaCa-2 cells to MET5A cells. Shown are monitoring at 4000Hz. MET5A^Control^, PANC1^Control^, MIAPaCa2^Control^: control transfected cells; MET5A^ALCAM-KD^, PANC1^ALCAM-KD^: cells with ALCAM knockdown by way of cell transfection; MIAPaCa2^ALCAM-OE^: cells with ALCAM overexpression by way of cell transfection. Replicate *n* = 4.

**Figure 6:**
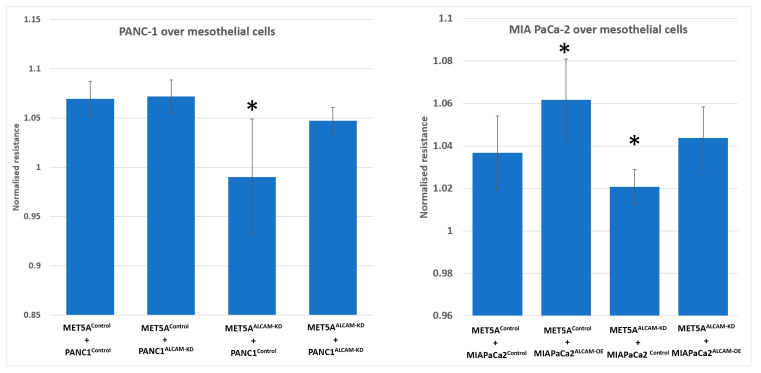
Interaction between PANC-1 (**left**) and MIA PaCa-2 (**right**) pancreatic cancer cells and MET5A mesothelial cells. Left: PANC-1 cells after knocking down ALCAM by way of knocking down showed reduced adhesion to MET5A mesothelial cells. Right: Overexpression of ALCAM in MIA PaCa-2 cells had augmented the interaction with mesothelial cells. * Groups of cells with ALCAM modification compared with groups of control MET5A cells plus control cancer cells (*p* < 0.05, replicate *n* = 4).

Similar to gastric cancer cells, we generated an ALCAM knockdown model from PANC-1 cell line which expressed high levels of ALCAM. In contrast, MIA PaCa-2 cells displayed low levels of ALCAM from which we created an ALCAM overexpression cell model. These created pancreatic cancer cell models were tested in the ECIS assay in a similar fashion to that of gastric cancer cells. As shown in Figure 5A–D, knocking down ALCAM from both the mesothelial cell MET5A and pancreatic cancer cell PANC-1 rendered a marked reduction of cell adhesion, similar to that seen with gastric cancer cells. In addition, overexpression of ALCAM in MIA PaCa-2 cells generated some marginal increases in the adhesiveness to MET5A cells (Figure 5E,F). This marginal increase is likely due to the low levels of ALCAM in the control cells and that over-expression only marginally increased the adhesiveness on the basis of the existing ALCAM. MET5A knockdown groups, which interacted with MIA PaCa-2 control or overexpression cells, also showed reduced cell adhesion compared with MET5A control groups (Figure 5G,H). Figure 6 showed the resistance value of each ECIS experimental group 1 h after cancer cells were added. It is clear that the adhesion of pancreatic cancer ALCAM knockdown cells to mesothelial cells, control or ALCAM knockdown, was significantly reduced (Figure 6).

### 2.5. DiI Based Pancreatic Tumour-Mesothelial Cell Interactions

**Figure 7:**
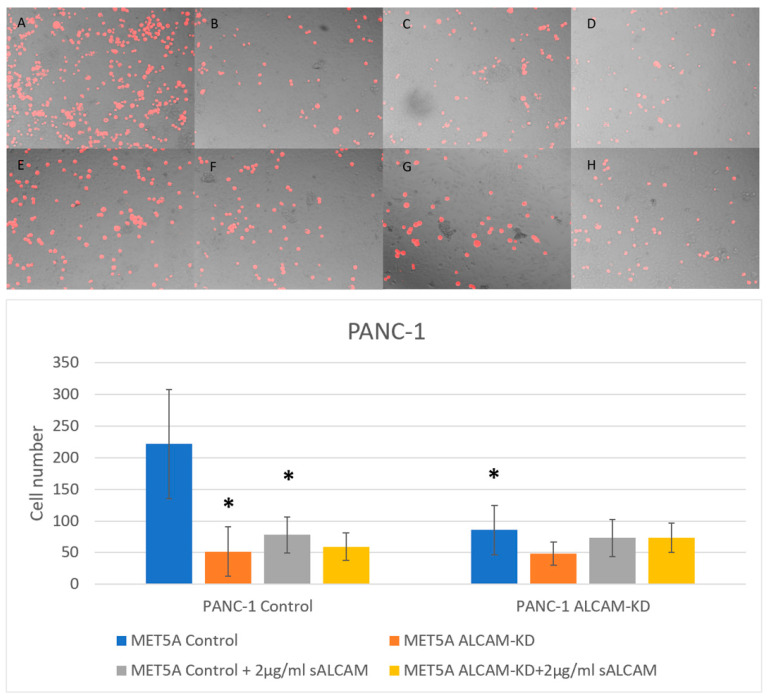
Interaction between pancreatic cancer cell line PANC-1 and mesothelial cell line MET5A as determined by the DiI based assays. (**Top**): Representative images (×10 magnification) of pancreatic cancer cells (PANC-1 Control and ALCAM knockdown cells) adherence to mesothelial cells (MET5A Control and ALCAM knockdown cells). (**A**–**D**) represent wells without any treatment and E-H represent wells treated with 2 µg/mL sALCAM. (**A**,**E**): MET5A Control + PANC-1 Control; (**B**,**F**): MET5A Control + PANC-1 ALCAM knockdown; (**C**,**G**): MET5A ALCAM knockdown + PANC-1 Control; (**D**,**H**): MET5A ALCAM knockdown + PANC-1 ALCAM knockdown. (**Bottom**): Graphical representation of pancreatic cancer cells (PANC-1 Control and ALCAM knockdown cells) adherence to mesothelial cells (MET5A Control and ALCAM knockdown cells). * Groups which showed significantly differences compared with “MET5A Control + PANC-1 Control” group (*p* < 0.05).

**Figure 8:**
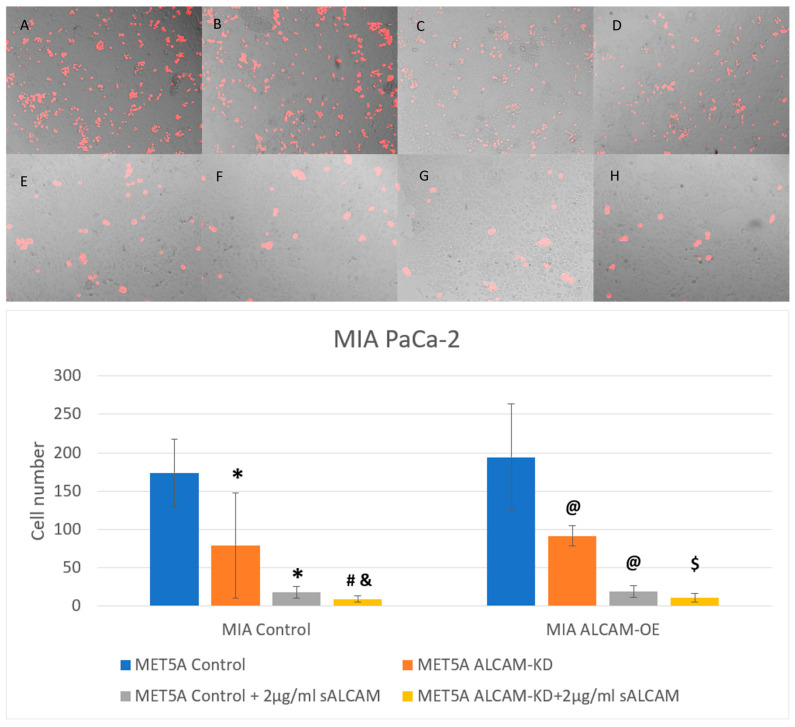
Interaction between pancreatic cancer cell line MIA PaCa-2 and mesothelial cell line MET5A as determined by the DiI based assays. (**Top**): Representative images (×10 magnification) of pancreatic cancer cells (MIA PaCa-2 Control and ALCAM overexpression cells) adherence to mesothelial cells (MET5A Control and ALCAM knockdown cells). (**A**–**D**) represent wells without any treatment and E-H represent wells treated with 2 µg/mL sALCAM. (**A**,**E**): MET5A control + MIA PaCa-2 control; (**B**,**F**): MET5A control + MIA PaCa-2 overexpression; (**C**,**G**): MET5A knockdown + MIA PaCa-2 control; (**D**,**H**): MET5A knockdown + MIA PaCa-2 overexpression. (**Bottom**): Graphical representation of pancreatic cancer cells (MIA Control and ALCAM overexpression cells) adherence to mesothelial cells (MET5A Control and ALCAM knockdown cells). *: Groups which showed significantly differences compared with “MET5A Control + MIA Control” group (*p* < 0.05); #: Groups which showed significantly differences compared with “MET5A ALCAM-KD + MIA Control” group (*p* < 0.05); &: Groups which showed significantly differences compared with” MET5A Control+ MIA Control + sALCAM” group (*p* < 0.05); @: Groups which showed significantly differences compared with “MET5A Control + MIA ALCAM-OE” group (*p* < 0.05); $: Groups which showed significantly differences compared with “MET5A ALCAM-KD + MIA ALCAM-OE” (*p* < 0.05).

With the findings from the dynamic interaction between tumour and mesothelial cells being mediated by ALCAM expression, we employed an end point analysis of tumour interactions by visualising this interaction at the end of the experiments. Here, we also used an antagonistic form of ALCAM, the soluble ALCAM known to compete with, and block the function of full membrane bound ALCAM, in order to analyze and confirm the observations seen with ECIS. Figure 7 shows the interaction between the pancreatic cancer cell line PANC-1 and mesothelial cell line MET5A. Loss of ALCAM in both mesothelial cells and pancreatic cancer cell line PANC-1 was able to ameliorate the adhesion of pancreatic cancer cells to mesothelial cells. This interaction could be blocked by sALCAM. On the other hand, MIA PaCa-2 cells, which expressed low levels of ALCAM, became more adhesive to the mesothelial cells following overexpression of ALCAM (Figure 8). Again, sALCAM showed a partial inhibition of the interactions.

### 2.6. DiI Based Gastric Tumour-Mesothelial Cell Interactions

**Figure 9:**
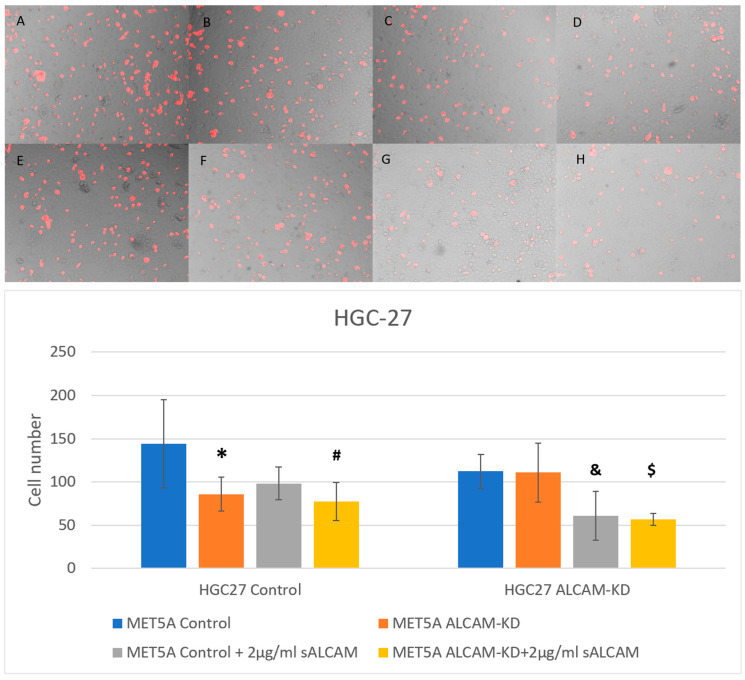
Interaction between gastric cancer cell line HGC-27 and mesothelial cell line MET5A as determined by the DiI based assays. (**Top**): Representative images (×10 magnification) of gastric cancer cells (HGC-27 Control and ALCAM knockdown cells) adherence to mesothelial cells (MET5A Control and ALCAM knockdown cells). (**A**–**D**) represent wells without any treatment and E-H represent wells treated with 2 µg/mL sALCAM. (**A**,**E**): MET5A Control + HGC27 Control; (**B**,**F**): MET5A Control + HGC27 ALCAM knockdown; (**C**,**G**): MET5A ALCAM knockdown + HGC27 Control; (**D**,**H**): MET5A ALCAM knockdown + HGC27 ALCAM knockdown. (**Bottom**): Graphical representation of gastric cancer cells (HGC-27 Control and ALCAM knockdown cells) adherence to mesothelial cells (MET5A Control and ALCAM knockdown cells). *: Groups which showed significantly differences compared with “MET5A Control + HGC27 Control” group (*p* < 0.05). #: Groups which showed significantly differences compared with “MET5A Control + HGC27 Control + sALCAM” group (*p* < 0.05); &: Groups which showed significantly differences compared with” MET5A Control+ HGC27 ALCAM-KD” group (*p* < 0.05); $: Groups which showed significantly differences compared with “MET5A ALCAM-KD + HGC27 ALCAM-KD” group (*p* < 0.05).

**Figure 10:**
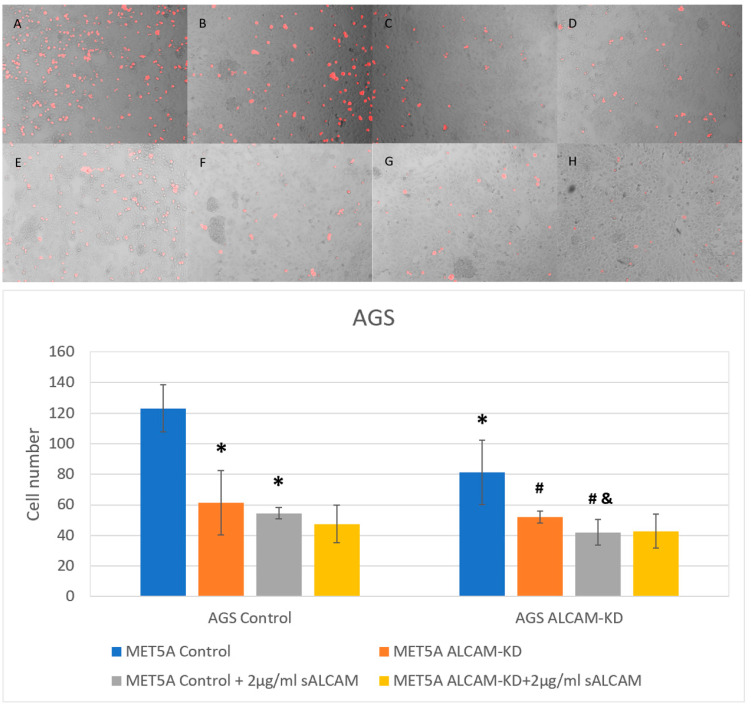
Interaction between gastric cancer cell line AGS and mesothelial cell line MET5A as determined by the DiI based assays. (**Top**): Representative images (×10 magnification) of gastric cancer cells (AGS Control and ALCAM knockdown cells) adherence to mesothelial cells (MET5A Control and ALCAM knockdown cells). (**A**–**D**) represent wells without any treatment and E-H represent wells treated with 2 µg/mL sALCAM. (**A**,**E**): MET5A control + AGS control; (**B**,**F**): MET5A control + AGS ALCAM knockdown; (**C**,**G**): MET5A ALCAM knockdown + AGS Control; (**D**,**H**): MET5A ALCAM knockdown + AGS ALCAM knockdown. (**Bottom**): Graphical representation of pancreatic cancer cells (AGS Control and ALCAM knockdown cells) adherence to mesothelial cells (MET5A Control and ALCAM knockdown cells). *: Groups which showed significantly differences compared with “MET5A Control + AGS Control” group (*p* < 0.05). #: Groups which showed significantly differences compared with “MET5A Control + AGS ALCAM-KD” group (*p* < 0.05); &: Groups which showed significantly differences compared with” MET5A Control + AGS Control + sALCAM” group (*p* < 0.05).

Similar to the pancreatic cell models, gastric cancer cell lines HGC-27 and AGS were also used to generate ALCAM knockdown cell models to examine tumour-mesothelial interaction together with ALCAM knockdown mesothelial cell line MET5A. As shown in Figure 9 and Figure 10, the number of adhered cells was reduced in MET5A cells with lower levels of ALCAM expression. Likewise, HGC-27 and AGS ALCAM knockdown cells showed reduced adhesiveness to MET5A cell monolayers. The minimum number of cells was observed in groups with both gastric ALCAM knockdown cells and mesothelial ALCAM knockdown cells. In addition, groups with sALCAM had significantly fewer cells adhered compared with those without.

### 2.7. The Interaction of Tumour-Mesothelial Cells Was Primarily Due to the Action of ALCAM and Mediated by the SRC Pathway

**Figure 11:**
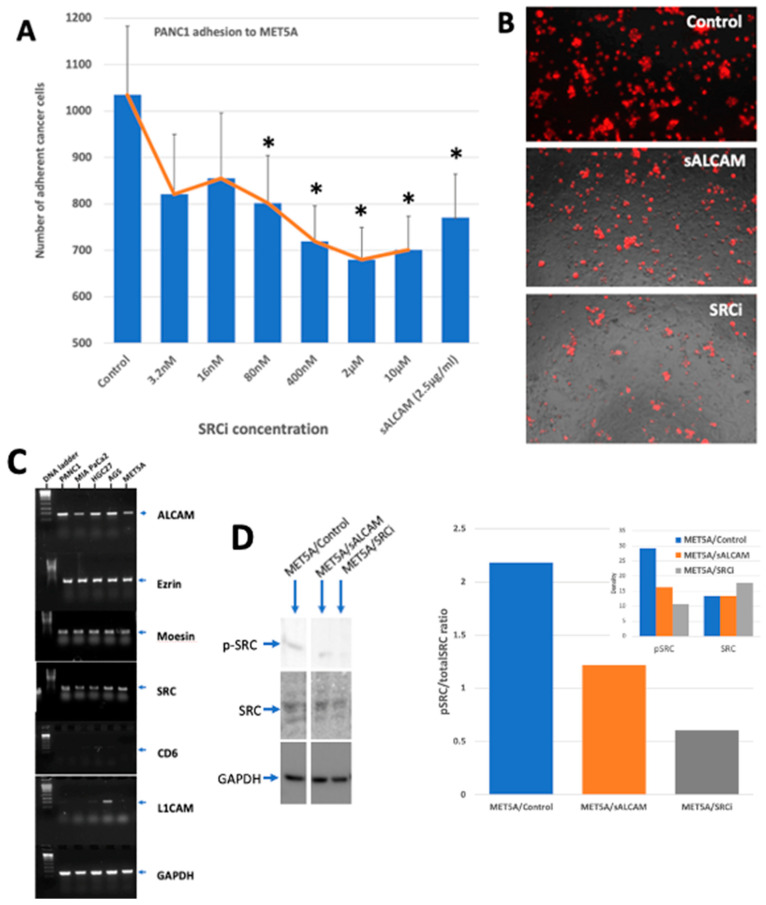
Tumour-mesothelial interaction and the role of SRC kinase. (**A**). The effects of SRC inhibitor (SRCi), AZM475271 on the interaction between pancreatic cancer cell MIA PaCa-2 and mesothelial cells by DiI assay. The SRC inhibitor suppressed the adhesion between 80 nM to 10 μM, to a degree similar to that of soluble ALCAM (* *p* < 0.05 versus control). (**B**). Representative images of tumour-mesothelial interaction. (**C**). Expression of potential ALCAM interacting partners in gastric and pancreatic cancer and mesothelial cells. All cells were negative for CD6 and L1CAM except that AGS was weakly positive for L1CAM (CD171). Cells were otherwise positive for SRC, and the ERM family ezrin and moesin. (**D**). SRC kinase expression and phosphorylation. MET5A cells were treated with soluble ALCAM (sALCAM) at 2.5 μg/mL or the SRC inhibitor (SRCi) AZM475271 at 400 nM for 40 min. Total SRC and phosphorylated-SRC (pSRC) was detected by protein blotting. Both sALCAM and SRCi inhibited the phosphorylation of SRC as shown in the bar graph. Insert: band density of respective SRC and p-SRC.

To explore if other key ALCAM-binding partners may contribute to the tumour-mesothelial interactions, we tested two well established partners, namely CD6 and L1CAM in all the cells used here. As shown in Figure 11, neither of the gastric, pancreatic or mesothelial cells expressed these two molecules (Figure 11C). We also evaluated the expression of the known subcoat protein and key signalling regulator for ALCAM, including the ERM family member and the SRC kinase. As shown in Figure 11, all 5 cell lines expressed high levels of ezrin, moesin and SRC gene transcript. These collectively suggest that the machinery for the ALCAM-ALCAM interaction in the cell model is intact. We next examine if the SRC kinase may contribute to the tumour-mesothelial interaction mediated by ALCAM seen here. First, we employed a SRC tyrosine kinase specific small inhibitor, AZM475271 in the tumour-mesothelial cell model. As shown in Figure 11B,C, there was a concentration dependent inhibition of tumour cell adhesion on mesothelial cells and at higher concentration, the AZM475271 achieved the same degree of inhibition as the soluble ALCAM antagonist. Both sALCAM and SRCi AZM475271 were able to inhibit the phosphorylation of SRC in the cell (Figure 11D).

## 3. Discussion

The present study has reported for the first time that levels of ALCAM in primary gastric cancer and primary pancreatic cancer has a significant value in predicting the likelihood of patients to develop peritoneal metastasis. The study has also revealed, for the first time that expression levels of ALCAM in mesothelial cells, the lining of the peritoneal cavity and cancer cells determines the degree of the adhesiveness of gastric and pancreatic cancer cells to mesothelial cells and that this interaction can be ameliorated by blocking the action of ALCAM, in this case, using a soluble ALCAM or a SRC kinase inhibitor. 

The mesothelium is the lining of the peritoneal cavity and consists of a monolayer of pavement-like mesothelial cells. Tumour cells are able to adhere to the peritoneum through either exposed extracellular matrix (ECM) or to the surface of mesothelial cells, where they exploit mesothelial cell surface receptors or the surface layer of hyaluronic acid to settle [47], followed consequently by adhesion, invasion and colony formation. It has been recognized in recent years that cancer cells shed from gastrointestinal tumours into the peritoneal cavity would adopt their cell surface CD44 to interact with hyaluronic acid (HA) synthesized by and overlaid the mesothelial cell layer of the cavity [33,34], to catalyse the seeding process over the mesothelial lining. This is one of the traditionally recognised ‘seed’ receptors on cancer cells to interact with the ‘soil’ receptor [35,48]. The findings presented here argue that ALCAM may function as both a ‘seed’ receptor (residing on gastric and pancreatic cancer cells) and a ‘soil’ receptor (residing on the receiving mesothelial cells) during the process of cancer cell seeding in the peritoneal cavity. It is reasonable to suggest that cancers cells, after shedding from GI cancers or ovarian cancers and falling into the peritoneal cavity, are going through the process of seeding and selection over the peritoneum. At this stage, the circulating cancer cells (seeds) would utilize the surface ALCAM to identify its homotypical partner on mesothelial cells and find the time and condition to settle over the peritoneum and form peritoneal surface tumours. Of course, this process is not alone for ALCAM since other known ‘seed and soil’ receptors would also participate in the tumour-mesothelial interaction. An example would be CD44 on the seeding cancer cells and hyaluronic acid on the surface of ‘soil’, the peritoneum. Certainly, in this interaction, one factor that has not been addressed by the present study and any other study is the flow force generated by the peristalsis of gastrointestinal organs on the settling of the seeding cancer cells over the soil peritoneum. This would make a highly useful addition to the understanding of how macroenvironment influences the seeding process in the microenvironment.

To further study the role of ALCAM in peritoneal metastasis, we generated cell models that express decreased or increased levels of ALCAM expression compared to controls. To monitor the impact of ALCAM alteration on the interaction of cancer and mesothelial cells, we performed both ECIS assay (that records the dynamic interaction between tumour and mesothelial cells) and DiI-based assay (measuring pancreatic/gastric tumour-endothelial interaction). Knocking down ALCAM in gastric or pancreatic cancer cells dramatically reduced the pace of adhesiveness to MET5A mesothelial cells that expressed high levels of ALCAM. On the other hand, MiaPaCa2 cells with low levels of ALCAM became more adhesive to the mesothelial cells following overexpression of ALCAM. These data support the argument that ALCAM in cancer cells plays a “seed receptor” role in the process of metastasis. In addition, knocking ALCAM from MET5A mesothelial cells resulted in a marginal reduction of adhesion by control cancer cells that express high levels of ALCAM, consistent with the argument that ALCAM in mesothelial cells has a role of the soil receptor for the tumour seeding ALCAM. Importantly, when ALCAM were knocked down from both cancer cells and mesothelial cells, the interaction between the two cell types were greatly reduced compared to an individual knocking down, further supporting the role of ALCAM in both “seed” and “soil” receptors during the process of metastasis. This finding on ALCAM has a similar echo to a recent study by Ng et al. [49] who reported that E-cadherin may have a similar property in an ex vivo gastric cancer model. Similar suggestions were indicated in the peritoneal metastasis from pancreatic cancer, although experimental data are yet to come [50]. 

ALCAM has been shown to act as a negative prognostic factor in multiple intra-abdominal tumours. A study by Ishigami et al. [51] showed that gastric cancer patients with positive membrane ALCAM staining had significantly shorter overall survival compared with negative staining patients. In a cohort study of pancreatic cancer cases, patients with higher levels of ALCAM was found to have significantly shorter overall survival, and pancreatic cancer tissues had significantly higher levels of ALCAM transcripts than normal pancreas tissues [41]. A meta-analysis demonstrated that high levels of ALCAM was correlated with shorter overall survival and the appearance of distant metastases in colorectal cancer [52]. 

One of the highlights of the present study is the finding that patients with high levels of ALCAM in gastric and pancreatic tumours tend to have a high chance of developing peritoneal metastasis. This provides a possible explanation of the negative prognostic effect of ALCAM in intra-abdominal tumours since peritoneal metastases have been recognised clinically as one of the most detrimental factors to influence the outcome of the patients. This clinical evidence supports the critical role of ALCAM in gastric/pancreatic cancer metastasis. Although the present study focused on gastric and pancreatic cancer, it is reasonable to assume that the same may well be seen in other tumours of the peritoneal cavity, including the non-functional ovarian cancers. More and larger size studies in this regard would be highly desirable.

The appearance of peritoneal metastases is naturally classified into stage IV, the final stage of cancers, at the worst in the classical TNM staging method. Since they cannot be diagnosed with only staging laparoscopy, this condition is termed the occult stage of peritoneal metastasis. When cancer cells adhere to and invade through the peritoneum and develop into colonies, the peritoneal metastasis progresses from the occult to the detectable stage. Collectively, early diagnosis is a challenge and effective treatment is also a significant medical and social challenge. We yet to develop effect way to manage peritoneal metastasis. Although intraperitoneal and systemic chemotherapies, along with other new approaches including immunotherapies have been practised in the treatment, the main intervention, the effectiveness, the unbearable side effects and the choice of options remain huge challenges [53]. These clearly call for the discovery of new ways to intervene the peritoneal metastasis. 

Previous studies have focused on the therapeutic implications of ALCAM in various cancer types. Wiiger et al. [54] have identified a single chain antibody (scFv173) of ALCAM. This ALCAM-targeted antibody was able to inhibit 50% of the invasion of breast cancer cells in an in vitro Matrigel invasion assay and reduce the growth rate of colorectal tumours in vivo. A study by Kinoshita and colleagues [55] showed that soluble ALCAM, in the name of extracellular soil signal sensor receptors (exSSSRs), could suppress the metastasis of lung cancer when it conjugated with a human IgG2-Fc. Sanders et al. [56] found out that a recombinant human ALCAM-Fc chimera was able to inhibit the adherence of prostate cancer cells to both endothelial cells and osteoblast cells.

Here, we have demonstrated that using a soluble form of ALCAM (sALCAM), the interaction between tumour cells and mesothelial cells was disrupted. Although this is not unique to the mesothelial interaction as the same as was reported in the tumour-endothelial interaction [41,56], it is nonetheless reasonable to suggest that the soluble form of sALCAM may be an option to consider in the peritoneal therapies and prevention when considering therapeutic option. This would make an exciting future study. Of course, other options to target ALCAM-ALCAM interactions including humanised antibodies to ALCAM, etc., could also be beneficial.

**Figure 12:**
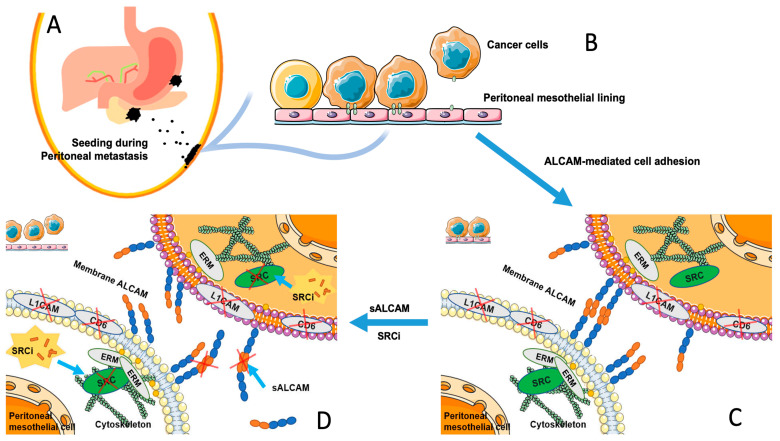
The proposed mechanism of ALCAM mediated tumour-endothelial interactions. Following shedding of cancer cells from the primary site, stomach or pancreas (**A**), the metastatic cancer cells (seeds) in the abdominal cavity come to contact the peritoneal mesothelial cells (‘soil’) (**B**), all expressed high levels of ALCAM but neither expressed CD6 or L1CAM (**C**), which initiates the ALACAM-ALCAM homotypic interaction. Supported by the machinery including the subcoat protein ERM and signalling kinase SRC, ALCAM mediates the tumour-mesothelial interaction. Soluble ALCAM as an extracellular antagonist or small compound inhibitory molecule to SRC kinase as intracellular inhibitor, can disrupt this ALCAM-ALCAM mediated tumour-endothelial interaction (**D**) and offer a potential therapeutic opportunity.

Finally, the present study has shown that the ‘seed’ and ‘soil’ interaction between cancer cells and the mesothelial cells is primarily mediated by ALCAM. The key known protein binding partners of ALCAM is ALCAM for the homotypic interaction, CD6 and L1CAM for heterotypic interactions at protein levels. It is well established that CD6 and L1CAM are seen largely in leukocytes and other immune cells and present as very low levels in other types of cells. Indeed, the present study has shown that all four cancer cell lines and the mesothelial cells are largely negative for CD6 and L1CAM. Supported by the evidence that ALCAM is strongly expressed in all cells used in the study and that the soluble ALCAM itself significantly inhibits the cell–cell interaction, it is collectively suggest that the tumour-mesothelial interactions seen here is mediated by ALCAM, namely the ALCAM-ALCAM homotypic protein interaction resulting in the heterotypic cancer-mesothelial cell interaction. However, it is likely that other cell adhesion molecules on the surface of the cancer cells and mesothelial cells may contribute to this interaction as a maximum effect by blocking ALCAM, by way of sALCAM and by genetic ALCAM knockdown are up to 60%. It has been established that ALCAM anchored to the cytoskeleton system of the cells via the ERM (ezrin-moesin-radixin) subcoat protein family and that this interaction is regulated intracellularly by the c-SRC kinase [57,58,59]. This mechanism is summarized in Figure 12.

## 4. Conclusions

ALCAM expression in gastrointestinal cancers, including gastric and pancreatic cancers, has a predictive value in the development of peritoneal metastasis, a detrimental condition for the patients. ALCAM proteins on cancer cells act as ‘seed’ receptor, and on peritoneal mesothelial cells function as a ‘soil’ receptor, through its homotypical protein–protein interactions. ALCAM-mediated tumour-mesothelial interaction leads to settling of cancer cells over the peritoneum, a process likely to require the participation of the SRC kinase signaling. Therefore, targeting ALCAM represents a novel therapeutic opportunity in both preventing and treating peritoneal metastases in gastric, pancreatic and other cancers.

## 5. Materials and Methods

### 5.1. Cell Lines and Cell Cultures

The human gastric cancer cell lines (AGS (ECACC ID 89090402) and HGC-27 (ECACC ID 94042256)) were purchased from ECACC (European Collection of Animal Cell Cultures, Salisbury, England, UK). Human pancreatic cancer cell lines (PANC-1 (CRL-1469) and MIA PaCa-2 (CRM-CRL-1420)) were obtained from the American Type Culture Collection (ATCC/LGC Standard, Teddington, England, UK). Human immortalized mesothelial cells, MET5A (CRL-9444) was also obtained from ATCC. Cells were cultured in a humidified incubator at 37 °C, 5% CO_2_ and 95% humidity in Dulbecco’s Modified Eagle Medium (DMEM)/F12 (Sigma-Aldrich, Dorset, England, UK) supplemented with 10% fetal Calf Serum (FCS) (Sigma-Aldrich, Dorset, UK) with 1% antibiotics (Sigma-Aldrich, Dorset, England, UK).

### 5.2. Key Reagents

Recombinant truncated human ALCAM-Fc chimera, containing ALCAM Trp28—Ala526 and a human IgG Fc region were purchased from R&D systems (Abingdon, England, UK) and is referred to as soluble ALCAM (sALCAM) here. The plasmids which contained shRNA targeting ALCAM and control plasmids containing scramble sequence were purchased from VectorBuilder Inc. (Chicago, IL, USA) as we previously reported [41,56]. The fluorescence dye, DiI (1,1′-Dioctadecyl-3,3,3′,3′Tetramethylindocarbocyanine Perchlorate), was purchased from Sigma-Aldrich (Dorset, England, UK). A monoclonal antibody to human ALCAM was purchased from Novocastra Laboratories Ltd (Milton Keynes, England, UK). Antibodies to c-SRC (SC-19) and phopho-c-SRC (SC-166860), and a housekeeping protein anti-GAPDH antibodies were from Santa Cruz Biotechnologies Inc. (Santa Cruz, CA, USA). A small inhibitor to human SRC, namely AZM475271 was from Tocris (Bristol, England, UK). 

### 5.3. Generation of ALCAM Modified Cells

Mesothelial cells (MET5A), pancreatic cancer cells (PANC-1 and MIA PaCa-2) and gastric cancer cells (AGS and HGC-27) were transfected with the plasmids in order to establish ALCAM-manipulated cell models. Fugene HD (Promega, Southampton, England, UK) transfection reagent was used in accordance with manufacturer’s instructions. It is a novel, nonliposomal transfection reagent designed to transfect DNA into a wide variety of cell lines with high efficiency and low toxicity. Following transfection, cells were subject to selection with 2 µg/mL puromycin (Fisher Scientific UK, Leicestershire, England UK), prepared in growth medium. Once sufficient cell death had occurred, cells were taken out of selection and grown routinely in maintenance medium containing 0.2 µg/mL puromycin.

### 5.4. Clinical Cohorts

A gastric cancer cohort and a pancreatic cancer cohort which were collected for the study were previously reported [41,60,61]. Following the same protocol both cohorts were collected immediately after surgical resection, under a local research ethics committee with patients’ consent. Patients were followed up for their clinical outcome and for the present study, and information on perineal metastases was recorded. In the pancreatic cohort, six patients were identified as having peritoneal metastasis and thirty five patients remained disease free during the following up period. In the gastric cancer cohort, eighteen patients developed peritoneal metastasis whilst one hundred and twelve patients remained disease free. The peritoneal metastasis group and disease free group were the subjects of comparison in the present study.

### 5.5. Determination of ALCAM in Tissues

The methods of evaluating the expression of ALCAM transcript has been previously reported [41,60,61]. RNA extraction was carried out with the TRI Reagent (Sigma-Aldrich, Dorset, England, UK). Tissue samples were prepared using a homogeniser (Cole Parmer, Cambridgeshire, England, UK) in the TRI Reagent. The RNA concentrations in cells and tissue samples were adjusted to the same and used to produce cDNA using a GoScript reverse transcription mix, Oligo (dT) kit (Promega, Southampton, UK) in accordance with the manufacturers’ guidelines. The levels of ALCAM in cells and tissues were determined by qPCR, with application of a molecular beacon based Amplifluor^TM^ Uniprimer^TM^ Universal qPCR system (Intergen Inc., Oxford, England, UK). The system was characterised by integrating a Z sequence (5′-ACTGAACCTGACCGTACA-3′) to the FAM-tagged Uniprimer^TM^ probe (Table 2). The reaction and detection were carried out using a StepOnePlus^TM^ Real-Time PCR System (Thermo Fisher Scientific, Leicestershire, England, UK). The amplification and detection conditions were: 95 °C for 10 min, 80 cycles of 95 °C for 10 sec, 55 °C for 35 sec (programmed for signal detection) and 72 °C for 10 sec. The transcripts were quantified alongside an internal standard to allow calculation of relative transcript copy numbers of the cells and tissues.

### 5.6. Tumour-Mesothelial Interaction Assay

The interaction between the two cell types was assessed using a method we previously described [62]. Briefly, the ALCAM-transfected cell models of gastric and pancreatic cancer were cultured to 60% to 80% confluence. The cell suspension was collected and stained with 5 µM DiI (1,1′-Dioctadecyl-3,3,3′,3′Tetramethylindocarbocyanine Perchlorate) for 30 min. After extensive washing to remove the free dyes, a fixed number of cells were added to mesothelial cell monolayer (MET5A control or ALCAM knockdown group), precoated to confluence on the floor of the 96-well plates. After 1 h, the culture wells were carefully washed with PBS to remove the non-adherent cancer cells. The remaining cells that adhered to the mesothelial cell monolayer were fixed with 4% formalin. Representative bright field and fluorescence images were captured on the EVOS fluorescent imaging system (EVOS FL Auto) (Life Technologies/ThermoFisher Scientific, Loghborough, England, UK) at 10 times objective magnification, and the images of merged and attached cancer cells were quantified by the automated counting tool of the building software of the EVOS system.

### 5.7. Dynamic Monitoring of Tumour-Mesothelial Interactions

In order to monitor the interactions between the two cell types, we employed an automated, multi-well and human interface-free method, namely the electric cell-substrate impedance sensing (ECIS) assay, a method to investigate cellular behaviour based on the impedance parameter detected from gold electrodes coated on the bottom of a 96-well array (Applied Biophysics Inc., Troy, NJ, USA) [63]. The assay to monitor cell interaction was modified according to the previously descried method [41,64]. In brief, prior to cell seeding, ECIS arrays containing growth medium were first stabilised using the stabilisation function within the system, allowing the gold surface of the electrode receptable to cells. Following by washing the arrays, mesothelial cells (control and ALCAM-modified) were seeded at an appropriate density before the 96-well array was equipped in the incubated array station and measured changes in resistance/impedance. Control and ALCAM-modified cancer cells were then added to the confluent monolayers of mesothelial cells. The cells responses were immediately and continuously monitored over a frequency of 4000 Hz. 

### 5.8. Protein Preparation and Protein Electrophesis

Cells were collected using a rubber cell scraper and pelleted by centrifugation. Added to the cell pellet was a lysis buffer contained NP40. Following extraction and centrifugation at 15,000 rpm at 4 °C, the insoluble were removed. Protein samples were added samples buffer and boiled at 100 °C. Equal amounts of protein from each sample were added to a Sodium dodecyl sulfate-polyacrylamide (SDS-PAGE, 8%) gel. The gel was subject to electrophoresis to separate proteins based on mass at 120 V, 50 W and 50 mA until sufficient separation was obtained. The gel was then transferred to an Immobilon-P PVDF membrane (Merck Millipore, Hertfordshire, UK) to perform semi-dry transfer via a semi-dry blotter at 15 V, 500 mA, 20 W for 45 min.

The membrane was blocked with 10% skimmed milk mixture diluted in Tris buffered saline (TBS) (Sigma-Aldrich Co, Poole, Dorset, UK) with 0.1% Tween-20 (Melford Laboratories Ltd., Suffolk, England, UK) for an hour before incubating with the appropriate primary overnight at 4 °C. The membrane was washed in 3% milk mixture three times for 15 min at room temperature then was incubated with secondary antibody conjugated with horses raddish peroxidise (HRP) (Sigma-Aldrich Co, Poole, Dorset, England, UK), which was diluted 1000 times in 3% milk mixture, for an hour at room temperature. After incubation, membrane was washed with TBS-T and TBS twice for 10 min. EZ-ECL solution (equal parts of solution A mix with solution B) (Geneflow Ltd., Litchfield, England, UK) was used to incubate with membrane in the dark before capturing images on a G-BOX (Syngene, Cambridge, England, UK) detection system. Protein band quantitation was carried out using ImageJ software (https://imagej.nih.gov, downloaded 15th December 2021) and is shown here as the total band volume. In the case of phosphorylation of SRC, the ratio between phosphorylated SRC and total SRC was also shown. 

### 5.9. Statistical Methods

SPSS (version 27) (IBM Corp. in Armonk, NY, USA) was used in all the statistical analyses. Pairwise comparisons were made by Student t-test. Survival analysis was by Kaplan–Meier survival analysis with log ranked comparison. Cox regression model was used to analyse the hazard ratio with the survival. *p* < 0.05 was regarded as statistically significant. 

## Figures and Tables

**Table 1:** ALCAM and patient’s peritoneal metastasis-free survival in gastric and pancreatic cancer.

	HR	P *	Survival Time
Gastric cancer	8.79	0.003	92.8 ± 2.3 vs. 80.2 ± 4.5 Months
Pancreatic cancer	2.84	0.208	93.1 ± 11.3 vs. 66.5 ± 15.3 Months

* By Cox regression model.

**Table 2:** Primers used in the study.

Target	Forward Primer	Reverse Primer *
ALCAM	ttatcataccttgccgatt	gggtggaagtcatggtatag
ALCAM	caggaggttgaaggactaaa	actgaacctgaccgtacagggatcagttttctttgtca
CD6	ctactgcggccacaaag	actgaacctgaccgtacactcggaagtgtacctcca
L1CAM	ccacttgtttaaggagagga	actgaacctgaccgtacagatgatggcactcacaaag
SRC	tgtggccctctatgactatg	aaactccccttgctcatgta
Ezrin	tggagagagagaaagagcag	ttcttctctgcctcagtgat
Moesin	taagaaggctcagcaagaac	cttcttggactcatctctgg
GAPDH	ggctgcttttaactctggta	gactgtggtcatgagtcctt
GAPDH	aaggtcatccatgacaactt	actgaacctgaccgtacagccatccacagtcttctg

* Sequence underlined are the Z-sequence for QPCR reaction.

## Data Availability

Data from the present study can be obtained from the authors upon reasonable request.
